# Longitudinal Trends in Body Mass Index Before and During the COVID-19 Pandemic Among Persons Aged 2–19 Years — United States, 2018–2020

**DOI:** 10.15585/mmwr.mm7037a3

**Published:** 2021-09-17

**Authors:** Samantha J. Lange, Lyudmyla Kompaniyets, David S. Freedman, Emily M. Kraus, Renee Porter, DNP, Heidi M. Blanck, Alyson B. Goodman

**Affiliations:** ^1^Division of Nutrition, Physical Activity, and Obesity, National Center for Chronic Disease Prevention and Health Promotion, CDC; ^2^Public Health Informatics Institute, Atlanta, Georgia; ^3^McKing Consulting Corporation, Atlanta, Georgia.

Obesity is a serious health concern in the United States, affecting more than one in six children (*1*) and putting their long-term health and quality of life at risk.* During the COVID-19 pandemic, children and adolescents spent more time than usual away from structured school settings, and families who were already disproportionally affected by obesity risk factors might have had additional disruptions in income, food, and other social determinants of health.^†^ As a result, children and adolescents might have experienced circumstances that accelerated weight gain, including increased stress, irregular mealtimes, less access to nutritious foods, increased screen time, and fewer opportunities for physical activity (e.g., no recreational sports) (*2*,*3*). CDC used data from IQVIA’s Ambulatory Electronic Medical Records database to compare longitudinal trends in body mass index (BMI, kg/m^2^) among a cohort of 432,302 persons aged 2–19 years before and during the COVID-19 pandemic (January 1, 2018–February 29, 2020 and March 1, 2020–November 30, 2020, respectively). Between the prepandemic and pandemic periods, the rate of BMI increase approximately doubled, from 0.052 (95% confidence interval [CI] = 0.051–0.052 to 0.100 (95% CI = 0.098–0.101) kg/m^2^/month (ratio = 1.93 [95% CI = 1.90–1.96]). Persons aged 2–19 years with overweight or obesity during the prepandemic period experienced significantly higher rates of BMI increase during the pandemic period than did those with healthy weight. These findings underscore the importance of efforts to prevent excess weight gain during and following the COVID-19 pandemic, as well as during future public health emergencies, including increased access to efforts that promote healthy behaviors. These efforts could include screening by health care providers for BMI, food security, and social determinants of health, increased access to evidence-based pediatric weight management programs and food assistance resources, and state, community, and school resources to facilitate healthy eating, physical activity, and chronic disease prevention.

Data were obtained from IQVIA’s Ambulatory Electronic Medical Records database,^§^ which contains deidentified information recorded during outpatient encounters for a geographically diverse U.S. patient population. BMI was calculated from height and weight measurements^¶^ and categorized based on sex-specific CDC BMI-for-age percentiles.** To be included, persons had to be aged 2–19 years at their initial BMI measurement and have two or more BMI measurements before the COVID-19 pandemic (with at least one during the year immediately preceding the pandemic, March 1, 2019–February 29, 2020) and one or more BMI measurements after the initial 3 months of the pandemic (June 1, 2020–November 30, 2020).^††^ The longitudinal cohort included 432,302 persons who had a total of 2.5 million BMI measurements collected from January 1, 2018 through November 30, 2020.

Linear mixed-effects regression models were used to examine differences in the average monthly rate of change in BMI before and during the COVID-19 pandemic. Models accounted for all BMI measurements for each child during the study period and included random intercepts to account for individual-level heterogeneity. Models included a linear time trend (from the start of the pandemic on March 1, 2020), a dichotomous variable designating BMI measurements to the period before or after the start of the pandemic on March 1, 2020, the interaction between the linear time trend and pandemic variable, sex (male or female), age (on March 1, 2020), race and ethnicity (White, Black, Asian, Hispanic, other, or unknown), and initial BMI category (underweight, healthy weight, overweight, moderate obesity, or severe obesity). Models were run on the full cohort and stratified by age group during the pandemic (3–5, 6–11, 12–17, and 18–20 years). Models were also calculated with weight change (pounds per month) and obesity status (BMI ≥95th percentile) as the outcomes.^§§^

To determine changes between the prepandemic and pandemic periods, CDC calculated rate differences as the pandemic slope minus prepandemic slope and rate ratios as the pandemic slope divided by prepandemic slope. Data were analyzed using SAS (version 9.4; SAS Institute Inc.) and Stata (version 15.1; StataCorp); statistical significance was defined as p<0.05. This activity was reviewed by CDC and conducted consistent with applicable federal law and CDC policy.^¶¶^

Among 432,302 persons aged 2–19 years in the longitudinal cohort, 50.7% were male and 65.7% were White (Table 1). The cohort included 45.7% persons from the South, 21.2% from the Midwest, 19.0% from the West, and 14.0% from the Northeast U.S. Census regions.*** Based on initial BMI, obesity prevalence was 16.1%, including 4.8%with severe obesity. Overall, the monthly rate of BMI increase nearly doubled during the COVID-19 pandemic period compared with that during the prepandemic period (0.100 versus 0.052 kg/m^2^; ratio = 1.93) (Table 2). Similarly, the rate of change in the proportion of persons with obesity was 5.3 times as high during the pandemic (0.37 percentage points per month) than before the pandemic (0.07); for example, in this cohort, the estimated proportion of persons aged 2–19 years with obesity was 19.3% (95% CI = 19.1–19.4) in August 2019 and 22.4% (95% CI = 22.3–22.6) in August 2020.

**TABLE 1:** Characteristics of the longitudinal cohort* of persons aged 2–20 years (N = 432,302) and those with at least one body mass index measurement in the year preceding the COVID-19 pandemic but not during the pandemic — IQVIA Ambulatory Electronic Medical Records Database, United States, January 2018─November 2020

Characteristic	No. (%)	
	Persons aged 2–20 years in the IQVIA longitudinal cohort*	Persons aged 2–20 years in the IQVIA database with ≥1 BMI measurement in the year preceding but not during the pandemic
**Total**	**432,302 (100.0)**	**1,419,796 (100.0)**
**Sex**		
Female	213,303 (49.3)	717,568 (50.5)
Male	218,999 (50.7)	702,228 (49.5)
**Race/Ethnicity^†^**		
White	283,915 (65.7)	840,906 (59.2)
Black	41,466 (9.6)	135,758 (9.6)
Asian	12,427 (2.9)	39,186 (2.8)
Hispanic	4,203 (1.0)	18,001 (1.3)
Unknown	72,010 (16.7)	325,809 (22.9)
Other	18,281 (4.2)	60,136 (4.2)
**Age group, yrs** ^§^		
2–5	106,944 (24.7)	284,872 (20.1)
6–11	155,389 (35.9)	407,720 (28.7)
12–17	144,302 (33.4)	487,031 (34.3)
18–20	25,667 (5.9)	240,173 (16.9)
**Initial BMI category^¶^**		
Underweight	18,293 (4.2)	58,801 (4.1)
Healthy weight	279,351 (64.6)	877,775 (61.8)
Overweight	65,281 (15.1)	221,749 (15.6)
Obesity	69,377 (16.0)	261,471 (18.4)
Moderate	48,715 (11.3)	172,206 (12.1)
Severe	20,662 (4.8)	89,265 (6.3)
Geographic region**^,^**^††^**		
South	197,639 (45.7)	696,998 (49.1)
Northeast	60,677 (14.0)	158,036 (11.1)
Midwest	91,704 (21.2)	275,896 (19.4)
West	82,173 (19.0)	288,244 (20.3)

**Abbreviation:** BMI = body mass index.

*The longitudinal cohort included persons aged 2–19 years at initial BMI measurement, with ≥2 BMI measurements before the pandemic (with ≥1 measurement during the year immediately preceding the pandemic) and ≥1 BMI measurement after the initial 3 months of the pandemic.

**^†^** Race and ethnicity categories are mutually exclusive. IQVIA’s Ambulatory Electronic Medical Records database lacks additional information on race and ethnicity because of information being optionally reported in a single composite variable in the electronic health record.

^§^ Based on age in years on March 1, 2020 (the start of the COVID-19 pandemic period for this analysis). Persons were aged 2–19 years at their initial BMI measurement and aged 3–20 years by March 1, 2020.

**^¶^** Based on initial BMI measurement. BMI categories were defined as underweight (<5th percentile), healthy weight (≥5th to <85th percentile), overweight (≥85th to <95th percentile), moderate obesity (≥95th percentile to <120% of the 95th percentile), and severe obesity (≥120% of the 95th percentile). Moderate obesity and severe obesity are mutually exclusive.

** A total of 109 persons in the longitudinal cohort and 622 persons with one or more BMI measurements in the year preceding but not during the pandemic were missing information on geographic region.

**^††^** U.S. Census regions: *Northeast*: Connecticut, Maine, Massachusetts, New Hampshire, New Jersey, New York, Pennsylvania, Rhode Island, and Vermont; *Midwest*: Illinois, Indiana, Iowa, Kansas, Michigan, Minnesota, Missouri, Nebraska, North Dakota, Ohio, South Dakota, and Wisconsin; *South*: Alabama, Arkansas, Delaware, District of Columbia, Florida, Georgia, Kentucky, Louisiana, Maryland, Mississippi, North Carolina, Oklahoma, South Carolina, Tennessee, Texas, Virginia, and West Virginia; *West*: Alaska, Arizona, California, Colorado, Hawaii, Idaho, Montana, Nevada, New Mexico, Oregon, Utah, Washington, and Wyoming.

**TABLE 2:** Monthly rate of change in the body mass index and weight of persons aged 2–19 years before and during the COVID-19 pandemic, overall and by body mass index category and age group — IQVIA Ambulatory Electronic Medical Records Database, United States, January 2018─November 2020

Characteristic	Prepandemic	Pandemic	Pandemic versus prepandemic	
	Slope* (95% CI)	Slope (95% CI)	Difference^†^ (95% CI)	Ratio^§^ (95% CI)
**BMI (kg/m^2^)**				
**Overall**	**0.052 (0.051 to 0.052)**	**0.100 (0.098 to 0.101)**	**0.05 (0.05 to 0.05)**	**1.93 (1.90 to 1.96)**
**Initial BMI category¶**				
Underweight	0.046 (0.044 to 0.047)	0.051 (0.044 to 0.058)	0.01 (0.00 to 0.01)	1.12 (0.96 to 1.28)
Healthy weight	0.044 (0.044 to 0.044)	0.078 (0.076 to 0.080)	0.03 (0.03 to 0.04)	1.78 (1.73 to 1.82)
Overweight	0.057 (0.056 to 0.058)	0.121 (0.117 to 0.125)	0.06 (0.06 to 0.07)	2.13 (2.06 to 2.20)
Moderate obesity	0.070 (0.069 to 0.071)	0.164 (0.160 to 0.168)	0.09 (0.09 to 0.10)	2.34 (2.28 to 2.40)
Severe obesity	0.089 (0.088 to 0.090)	0.179 (0.173 to 0.185)	0.09 (0.08 to 0.10)	2.00 (1.93 to 2.07)
Age group, yrs**				
3–5	−0.002 (−0.003 to −0.002)	0.040 (0.037 to 0.043)	0.04 (0.04 to 0.05)	—††
6–11	0.059 (0.059 to 0.060)	0.148 (0.145 to 0.150)	0.09 (0.09 to 0.09)	2.50 (2.45 to 2.54)
12–17	0.072 (0.071 to 0.072)	0.106 (0.104 to 0.109)	0.03 (0.03 to 0.04)	1.48 (1.44 to 1.51)
18–20	0.045 (0.044 to 0.046)	0.032 (0.027 to 0.037)	−0.01 (−0.02 to −0.01)	0.70 (0.59 to 0.82)
**Weight, lbs**				
**Overall**	**0.356 (0.354 to 0.358)**	**0.595 (0.588 to 0.603)**	**0.24 (0.23 to 0.25)**	**1.67 (1.65 to 1.69)**
**Initial BMI category**				
Underweight	0.212 (0.205 to 0.218)	0.289 (0.252 to 0.325)	0.08 (0.04 to 0.11)	1.36 (1.19 to 1.54)
Healthy weight	0.282 (0.280 to 0.284)	0.447 (0.438 to 0.457)	0.17 (0.16 to 0.18)	1.59 (1.55 to 1.62)
Overweight	0.409 (0.405 to 0.412)	0.725 (0.706 to 0.744)	0.32 (0.30 to 0.34)	1.78 (1.73 to 1.82)
Moderate obesity	0.544 (0.541 to 0.548)	1.010 (0.989 to 1.032)	0.47 (0.44 to 0.49)	1.86 (1.81 to 1.90)
Severe obesity	0.736 (0.730 to 0.741)	1.217 (1.187 to 1.248)	0.48 (0.45 to 0.51)	1.65 (1.61 to 1.70)
**Age group, yrs**				
3–5	0.379 (0.374 to 0.383)	0.469 (0.453 to 0.485)	0.09 (0.07 to 0.11)	1.24 (1.20 to 1.28)
6–11	0.365 (0.362 to 0.367)	0.737 (0.724 to 0.750)	0.37 (0.36 to 0.39)	2.02 (1.98 to 2.06)
12–17	0.393 (0.391 to 0.396)	0.623 (0.609 to 0.636)	0.23 (0.22 to 0.24)	1.58 (1.55 to 1.62)
18–20	0.277 (0.272 to 0.282)	0.202 (0.174 to 0.229)	−0.08 (−0.10 to −0.05)	0.73 (0.63 to 0.83)

**Abbreviations:** BMI = body mass index; CI = confidence interval.

* Measured in kg/m^2^/month for BMI analysis, pounds per month for weight analysis.

^†^ Calculated as the pandemic slope minus prepandemic slope. Units are kg/m^2^ per month; 95% CIs for differences that exclude the null value of 0 are statistically significant.

^§^ Calculated as the pandemic slope divided by prepandemic slope; 95% CIs for ratios that exclude the null value of 1 are statistically significant.

^¶^ Based on initial BMI measurement. BMI categories were defined as underweight (<5th percentile), healthy weight (≥5th to <85th percentile), overweight (≥85th to <95th percentile), moderate obesity (≥95th percentile to <120% of the 95th percentile), and severe obesity (≥120% of the 95th percentile). Mixed-effects model included sex, race and ethnicity, age in years on March 1, 2020, and threeway interaction among linear time trend, pandemic indicator variable, and BMI category. Model for weight in pounds also included height in inches and height squared.

** Based on age in years on March 1, 2020, which was the start of the COVID-19 pandemic for this analysis. Persons were aged 2–19 years at their initial BMI measurement and aged 3–20 years by March 1, 2020. Mixed-effects model included sex, race and ethnicity, age in years on March 1, 2020, initial BMI category, and threeway interaction among linear time trend, pandemic indicator variable, and age group. Model for weight in pounds also included height in inches and height squared.

^††^ Ratio was not calculated because of a prepandemic slope that was negative and very close to zero.

Persons aged 2–19 years in all BMI categories except underweight experienced significant increases in their rate of BMI change during the pandemic (Table 2). Among persons with overweight, moderate obesity, and severe obesity, pandemic rates of BMI increase more than doubled, compared with prepandemic rates (ratios = 2.13, 2.34, and 2.00; differences = 0.06, 0.09, and 0.09, respectively); similar effects were observed for weight change. In contrast, those with healthy weight had a rate of BMI change that increased 0.03 kg/m^2^/month during the pandemic (ratio = 1.78).

Compared with other age groups, children aged 6–11 years experienced the largest increase in their rate of BMI change (0.09 kg/m^2^/month), with a pandemic rate of change that was 2.50 times as high as the prepandemic rate. Age-stratified analyses revealed that among children aged 3–5 and 6–11 years, the difference in the rate of BMI change increased with increasing BMI category. For example, among children aged 3–5 years, those with healthy weight had an increase in their rate of BMI change of 0.03 kg/m^2^/month, whereas those with overweight, moderate obesity, or severe obesity had increases of 0.06, 0.10, and 0.18 kg/m^2^/month, respectively (Figure).

**FIGURE:**
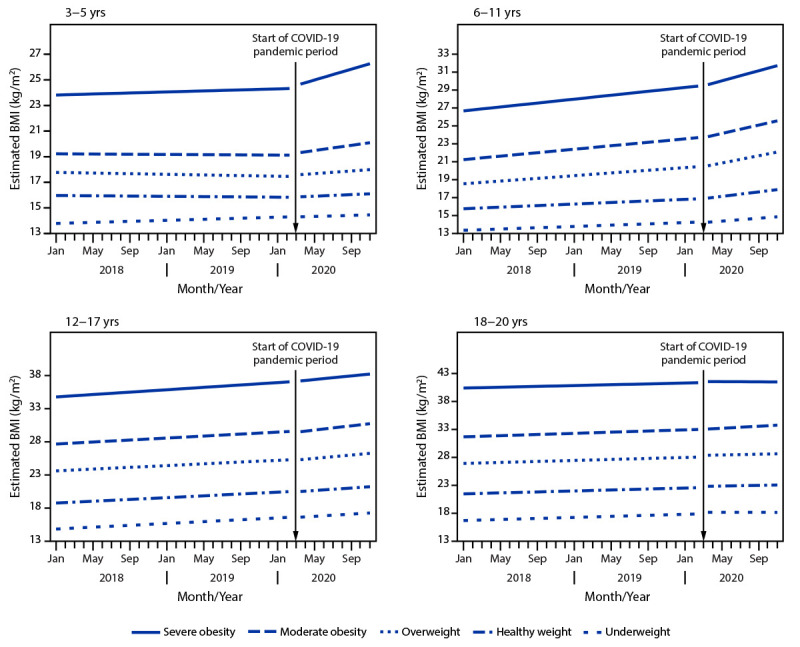
Estimated body mass index before and during the COVID-19 pandemic, by initial body mass index category, stratified by age group — IQVIA Ambulatory Electronic Medical Records Database, United States, January 2018–November 2020 **Abbreviation:** BMI = body mass index.

## Discussion

In a longitudinal cohort of 432,302 persons aged 2–19 years with outpatient visits, the monthly rate of increase in BMI nearly doubled during the COVID-19 pandemic compared with a prepandemic period. The estimated proportion of persons aged 2–19 years with obesity in this care-seeking cohort also increased during the pandemic; for example, 19.3% of persons had obesity in August 2019 compared with 22.4% 1 year later. These findings are consistent with a recent study of Kaiser Permanente data that reported significant weight gain and increased obesity prevalence during the pandemic among children and adolescents aged 5–17 years in Southern California (*4*). The present study is the largest and first geographically diverse analysis to assess the association of the COVID-19 pandemic with BMI and the first to show results by initial BMI category.

Persons aged 2–19 years with moderate or severe obesity before the pandemic experienced significantly higher rates of increase in BMI, which translates to weight gain, compared with those with prepandemic healthy weight. During March–November 2020, persons with moderate or severe obesity gained on average 1.0 and 1.2 pounds per month, respectively. Weight gain at this rate over 6 months is estimated to result in 6.1 and 7.6 pounds, respectively, compared with 2.7 pounds in a person with healthy weight. Accelerated weight gain, especially among children with overweight or obesity, can cause long-lasting metabolic changes that put children at risk for serious and costly co-occurring conditions, such as type 2 diabetes, hypertension, and depression (*5*,*6*).

In response to pandemic-related concerns and because of the critical role that pediatricians serve in maintenance of healthy child weight (*7*), the American Academy of Pediatrics recommended that pediatricians assess all children for the onset of obesity-related risk factors during the pandemic and provide tailored counseling, including screening for patient and family stress, disordered eating, and social determinants of health.^†††^ The large increases in BMI and weight detailed in this report provide additional support for the need for such comprehensive screening and counseling.

Consistent with previous studies (*4*,*8*), this analysis found that preschool and school-aged children, particularly those with obesity, had larger pandemic-associated increases in BMI than did adolescents. During the pandemic, many early child care and education settings^§§§^ and schools^¶¶¶^ experienced closures, leading to online or hybrid learning environments. This might have reduced the ability for some children to engage in structured physical activity and receive healthy meals. As venues serving youth reopen, it is important to acknowledge the potential indirect consequences of the pandemic and provide children, adolescents, and families with ample opportunities for proper nutrition and regular physical activity.

The findings in this report are subject to at least five limitations. First, although the longitudinal cohort included a geographically diverse sample of persons with clinically measured BMI data, IQVIA data are not nationally representative; this analysis should be replicated with other data sets, particularly those that are population-based. Second, IQVIA lacks detailed data on race and ethnicity because information was optionally reported in a single composite variable; therefore, ability to assess outcomes by racial and ethnic subpopulations was limited. Third, the number of health care visits with measured BMI was substantially lower during the beginning of the pandemic (March–May 2020) than during comparable months in 2018 and 2019, suggesting potential selection bias for persons who sought health care in 2020; to minimize bias, persons were required to have a BMI measurement during June–November 2020, when health care–seeking behavior began to normalize, to be included in the cohort. Fourth, the observed associations might represent an over- or underestimation if persons who gained weight during the pandemic were more or less likely to see a doctor because of health status or social determinants of health, such as access to care. Finally, the findings could be attributed to other factors that coincided with the pandemic dates selected for this study.

In this large, longitudinal cohort of persons aged 2–19 years, sharp increases in BMI rates occurred during the COVID-19 pandemic; those with overweight or obesity and younger school-aged children experienced the largest increases. These findings underscore the importance of obesity prevention and management efforts during and following the COVID-19 pandemic, as well as during future public health emergencies, including increased access to efforts that promote healthy behaviors. These efforts could include screening for BMI, food security, and other social determinants of health by health care providers; increased access to evidence-based pediatric weight management programs and food assistance resources; and state, community, and school efforts to facilitate healthy eating, physical activity, and chronic disease prevention.****

**Summary**

**What is already known about this topic?**

The COVID-19 pandemic led to school closures, disrupted routines, increased stress, and less opportunity for physical activity and proper nutrition, leading to weight gain among children and adolescents.

**What is added by this report?**

Among a cohort of 432,302 persons aged 2–19 years, the rate of body mass index (BMI) increase approximately doubled during the pandemic compared to a prepandemic period. Persons with prepandemic overweight or obesity and younger school-aged children experienced the largest increases.

**What are the implications for public health practice?**

Obesity prevention and management efforts during and following the COVID-19 pandemic could include health care provider screening for BMI, food security, and social determinants of health, and increased access to evidence-based pediatric weight management programs and food assistance resources.

## Footnotes

*
https://www.cdc.gov/obesity/childhood/causes.html

†
https://www.cdc.gov/socialdeterminants/about.html

§ Version 5, November 2020 data release. IQVIA's Ambulatory Electronic Medical Records database includes data for approximately 74 million persons from all 50 states treated by approximately 100,000 health care providers who are affiliated with approximately 800 ambulatory sites across the United States. The data set contains key clinical variables, including laboratory values, patient vitals, health behaviors, diagnoses, and procedures. All data were extracted using the E360 Software-as-a-Service Platform. https://www.iqvia.com/solutions/real-world-evidence/platforms/e360-real-world-data-platform

¶ Measured height and weight data during January 1, 2018–November 30, 2020 for persons aged 2–20 years in IQVIA were cleaned using growthcleanr (https://github.com/carriedaymont/growthcleanr), an open-source R package for cleaning pediatric growth data. Height and weight values were included if they were measured within 30 days of each other, resulting in data for 3,571,971 persons.

** CDC BMI-for-age percentiles were defined as underweight (<5th percentile), healthy weight (≥5th to <85th percentile), overweight (≥85th to <95th percentile), moderate obesity (≥95th percentile to <120% of the 95th percentile), and severe obesity (≥120% of the 95th percentile). https://www.cdc.gov/healthyweight/assessing/bmi/childrens_bmi/about_childrens_bmi.html

†† BMI measurements taken during the initial 3 months of the COVID-19 pandemic (March–May 2020) were included in the mixed effects models, as were all BMI measurements for the 432,302 persons in the longitudinal cohort; however, these measurements were not used to define cohort selection criteria. For example, if a child had a BMI measurement in March 2020, they had to have one in June–November 2020 to meet the “pandemic” BMI selection criterion and be cohort-eligible.

§§ The model with weight (pounds per month) as the outcome included height (inches) and height squared as additional covariates. The model with obesity status as the outcome was a generalized linear model with Poisson distribution and log link function.

¶¶ 45 C.F.R. part 46, 21 C.F.R. part 56; 42 U.S.C. Sect. 241(d); 5 U.S.C. Sect. 552a; 44 U.S.C. Sect. Sect. 3501 et seq.

*** IQVIA geographic regions align with U.S. Census regions. https://www.census.gov/prod/1/gen/95statab/preface.pdf

†††
https://www.aap.org/en/pages/2019-novel-coronavirus-covid-19-infections/clinical-guidance/obesity-management-and-treatment-during-covid-19/

§§§
https://www.cdc.gov/obesity/strategies/childcareece.html

¶¶¶
https://www.cdc.gov/healthyschools/index.htm

****
https://www.cdc.gov/nccdphp/dnpao/state-local-programs/index.html
