# *Erratum:* Vol.70, No. 37

**DOI:** 10.15585/mmwr.mm7038a6

**Published:** 2021-09-24

In the report “Longitudinal Trends in Body Mass Index Before and During the COVID-19 Pandemic Among Persons Aged 2–19 Years — United States, 2018–2020,” on page 1279, the third sentence of the first full paragraph should have read, “Based on initial BMI, obesity prevalence was **16.0**%, including 4.8% with severe obesity.” On page 1280, the last sentence should have read, “Weight gain at this rate over 6 months is estimated to result in 6.1 and **7.3** pounds, respectively, compared with 2.7 pounds in a person with healthy weight.” On page 1281, the figure title should have read, “Estimated body mass index before and during the COVID-19 pandemic, by initial body mass index category, stratified by age group — IQVIA Ambulatory Electronic Medical Records Database, United States, **January 2018–November 2020**.”

